# Increasing evapotranspiration decouples the positive correlation between vegetation cover and warming in the Tibetan plateau

**DOI:** 10.3389/fpls.2022.974745

**Published:** 2022-09-23

**Authors:** Xue Dai, Zhongbo Yu, Ashley M. Matheny, Wei Zhou, Jun Xia

**Affiliations:** ^1^State Key Laboratory of Hydrology-Water Resources and Hydraulic Engineering, Hohai University, Nanjing, China; ^2^Joint International Research Laboratory of Global Change and Water Cycle, Hohai University, Nanjing, China; ^3^College of Hydrology and Water Resources, Hohai University, Nanjing, China; ^4^Department of Geological Sciences, University of Texas at Austin, Austin, TX, United States; ^5^State Key Laboratory of Water Resources and Hydropower Engineering Science, Wuhan University, Wuhan, China

**Keywords:** vegetation cover, climate change, evapotranspiration, Tibetan Plateau, decoupling

## Abstract

Plant growth generally responds positively to an increase in ambient temperature. Hence, most Earth system models project a continuous increase in vegetation cover in the future due to elevated temperatures. Over the last 40 years, a considerable warming trend has affected the alpine ecosystem across the Tibetan Plateau. However, we found vegetation growth in the moderately vegetated areas of the plateau were negatively related to the warming temperatures, thus resulting in a significant degradation of the vegetative cover (LAI: *slope* = −0.0026 per year, *p* < 0.05). The underlying mechanisms that caused the decoupling of the relationship between vegetation growth and warming in the region were elaborated with the analysis of water and energy variables in the ecosystem. Results indicate that high temperatures stimulated evapotranspiration and increased the water consumption of the ecosystem (with an influence coefficient of 0.34) in these degrading areas, significantly reducing water availability (with an influence coefficient of −0.68) and limiting vegetation growth. Moreover, the negative warming effect on vegetation was only observed in the moderately vegetated areas, as evapotranspiration there predominantly occupied a larger proportion of available water (compared to the wet and highly vegetated areas) and resulted in a greater increase in total water consumption in a warmer condition (compared to dry areas with lower levels of vegetation cover). These findings highlight the risk of vegetation degradation in semi-arid areas, with the degree of vulnerability depending on the level of vegetation cover. Furthermore, results demonstrate the central role of evapotranspiration in regulating water stress intensity on vegetation under elevated temperatures.

## Introduction

The Tibetan Plateau, the largest geographical unit with the highest elevation on Earth, contains the largest ice masses outside the polar regions (Yao et al., 2012). Many prominent Asian rivers originate from the Tibetan Plateau; therefore, the plateau is characterised by extremely intense thermal erosion and erosion by water, wind, and ice (Yang et al., 2011; Yao et al., 2019). Consequently, vegetation in this harsh environment is vulnerable (with relatively low biodiversity, less primary productivity, and small vegetation coverage degree; Gao et al., 2016; Liu et al., 2018); yet, it provides remarkable ecosystem services such as surface soil conservation, water storage, and climate regulation (*via* evapotranspiration, carbon sequestration, and other key processes; Gao et al., 2016; Wang et al., 2018). Vegetation degradation in the Tibetan Plateau will induce substantial negative environmental consequences. These consequences can threaten the ecological security of both the plateau and its surrounding regions (Sun et al., 2016). Therefore, it is essential to monitor the status and tendencies of, and risk mitigation of the vegetation in the Tibetan Plateau.

In recent decades, the Tibetan Plateau has witnessed more rapid temperature changes than other regions globally (two-times higher warming rate than global average; Hu et al., 2019). Considering that the vegetation in the plateau faces substantial low temperature and drought stresses because of the cold and dry environments, warming trends have triggered considerable changes in the plateau’s vegetation (Zhang et al., 2017b). Even the response of alpine vegetation to climate change in the Tibetan Plateau has been discussed in lots of studies (Liu et al., 2018), there are still some controversies (Fan and Cheng, 2002; Pan et al., 2017; Piao et al., 2020) concerning the effects of warming on vegetation in this region. For instance, several studies have revealed that vegetation productivity in the Tibetan Plateau has increased to some extent in recent decades. This has been attributed to temperature increase and the subsequent reduction in low temperature stress (Fan and Cheng, 2002; Piao et al., 2020). However, some other studies have reported degradation of vegetation in many areas of the plateau (Sun et al., 2016; Pan et al., 2017). Although such degradation has been partially attributed to overgrazing in some areas, climate change was reported as one of the main reasons for the vegetation degradation. It is because the Tibetan Plateau is a nearly pristine natural ecosystem with a minimal footprint of human activity owing to its cold, dry, and hypoxic environment (Cuo et al., 2015; Deng and Zhang, 2018).

The aforementioned controversies emphasise the necessity for a more comprehensive study concerning warming influences on vegetation in the Tibetan Plateau. Moreover, the response of vegetation to warming—in nearly all terrestrial ecosystems—has become a widely discussed topic in recent climate-change impact studies (Medlyn et al., 2002; Zhu et al., 2016; Huang et al., 2019; Wang et al., 2019a, 2020). Specifically, previous empirical studies have reported a strong positive relationship between vegetation cover and growing season temperature (Berry and Bjorkman, 1980; Kattge and Knorr, 2007; Lloyd and Farquhar, 2008). The positive relationship has been incorporated in various terrestrial ecosystem models *via* mechanisms used to account for thermal acclimation—that is, a temporal adjustment of optimal photosynthetic rates to air temperature during growth (Smith et al., 2016; Mercado et al., 2018). However, some recent regional case studies have found a decoupling (or divergence) in the relationship between vegetation growth and warming (Medlyn et al., 2002; Matheny et al., 2015; Novick et al., 2016; Mercado et al., 2018; Merkle and Rosseel, 2018). For instance, D’Arrigo et al. (2004) reported a decline in the temperature sensitivity of tree growth during the late twentieth century, based on dendrochronological data. Research by Sanders-DeMott et al. (2020) revealed that the strength of the correlation between vegetation productivity and temperature declined from the early 1980s to 2011 in the northern hemisphere. Moreover, other studies found unimodal relationship curves between vegetation and temperature (Duffy et al., 2021). This implies that plant productivity increases with temperature up to a certain threshold and then decreases thereafter (Piao et al., 2014). These decoupling phenomena suggest that the hypothesis of a positive linear relationship between vegetation and temperature may overestimate the response of vegetation to climate warming. Furthermore, predictions on vegetation and temperature may even be incorrect considering these phenomena.

One classic hypothesis suggests that plants respond to rising temperature only for a short time (Fu et al., 2015). This is due to their ability to acclimatise to temperature changes, which begins to explain the mechanisms that underlie the decoupling phenomenon (Huntzinger et al., 2017; Zhao et al., 2020). The resource limitation hypothesis proposes that the decoupling response of vegetation depends on the interaction of multiple environmental resources and that the responsiveness of plants decreases with resource consumption (Angert et al., 2005; Beck and Goetz, 2011; Wu et al., 2012). Since water is a key resource for plant growth, some studies have tried to explain this decoupling through the lens of water transport mechanisms to explore the role of water in regulating plant response to temperature (Beier et al., 2012; Matheny et al., 2015; Parida et al., 2020; Liu et al., 2020b). It has been hypothesised that warming reduces water availability. For example, soil water content in the ecosystem is reduced through increasing evapotranspiration, which thereby inhibits plant growth (Medlyn et al., 2002; Cuo et al., 2015). Some studies suggest that stomatal closure triggered by the increase in vapour pressure deficit of air at elevated temperatures also inhibits vegetation growth (Sun et al., 2016). However, these feedbacks have not yet been tested systematically across ecosystems worldwide.

In this study, we systematically investigated long-term changes (1982–2020) in the degree of vegetation coverage in the Tibetan Plateau given the sensitivity and vulnerability of Tibetan Plateau’s vegetation to the warming temperatures. The response of vegetation coverage to temperatures in the plateau and the underlying mechanisms were also analysed, aiming to test whether the decoupling phenomenon exists and what processes drive it. Vegetation loss and a decoupling of vegetation growth response to warming in the moderately vegetated areas of the Tibetan Plateau were recorded. Integrative modelling using a structural equation model (SEM) attributed the decoupling phenomenon to warming-promoted evapotranspiration. This is due to evapotranspiration increasing water consumption in the ecosystem (with an influence coefficient of 0.34), thus increasing vegetation drought stress (with an influence coefficient of −0.68), and inhibiting vegetation growth. These findings overturn the hypothesis of a positive vegetation-temperature relationship in specific areas of the Tibetan Plateau. This also emphasises the risk of vegetation loss in other semi-arid areas with a certain degree of vegetation cover in response to increasing temperatures.

## Materials and methods

### Study area

**Figure 1:**
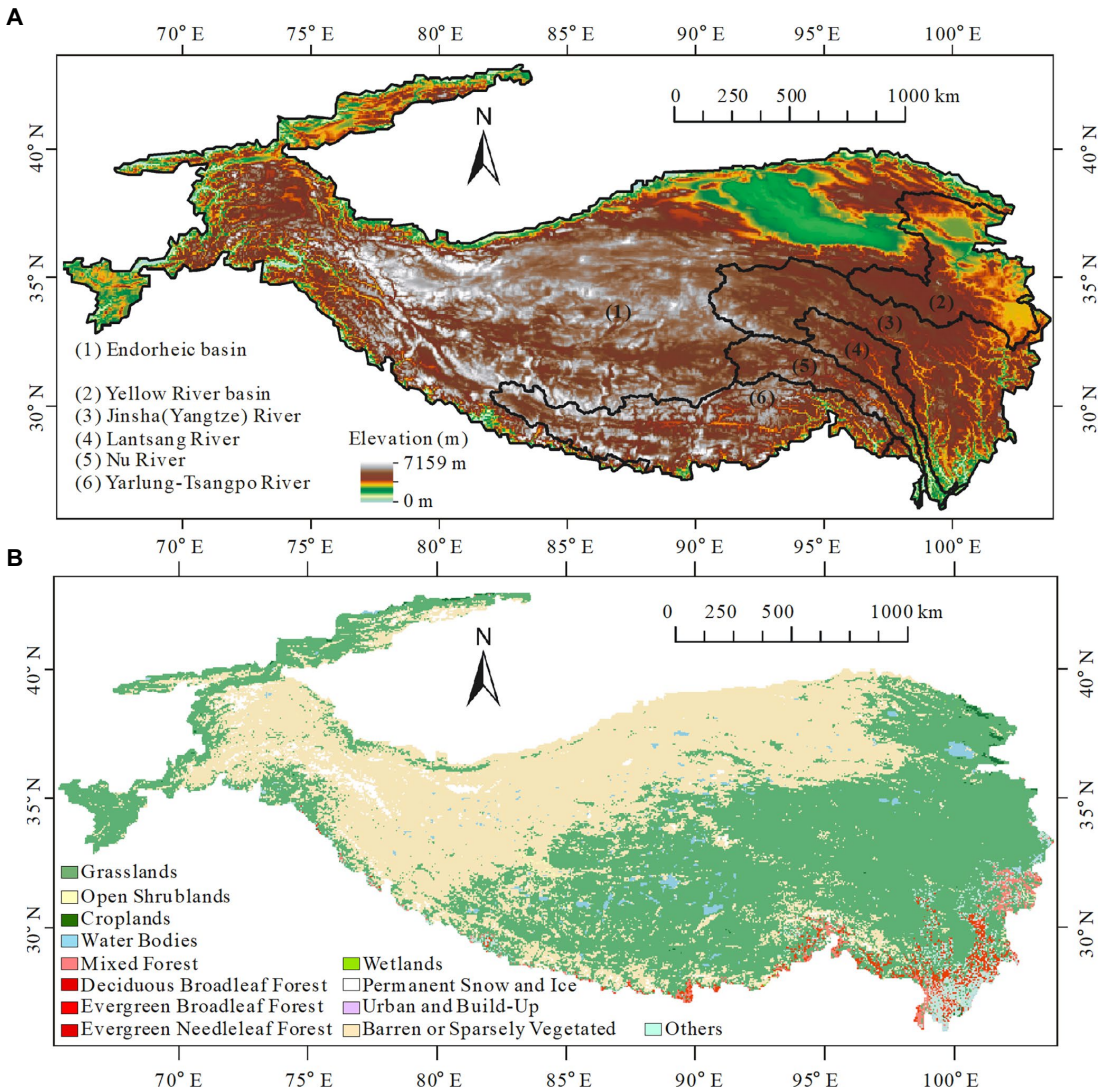
Basic topographical and vegetation conditions of the Tibetan Plateau. **(A)** The elevation gradients increasing towards the northwest. **(B)** International Geosphere-Biosphere Programme (IGBP) classification of the Tibetan Plateau (shown by the median of the IGBP maps during 2000–2020).

The Tibetan Plateau ranges from 66 to 104° E and 25 to 44° N (Figure 1) with a total area of over 2.5 million km^2^ (Yao et al., 2012, 2019). Surrounded by the high mountains of Asia, the plateau has an average altitude of more than 4,500 m above sea level, thus being called the “Roof of the World” and holding the biggest store of frozen water outside the poles (Wang et al., 2018). Inside the Tibetan Plateau, elevation increases towards the northwest while crossing mountain ranges (Figure 1A) substantially lowering the temperature and precipitation along a northwesterly transect. Consequently, the degree of vegetation cover follows a similar diminishing pattern. Vegetation types of the plateau also transit from forest to grassland and even to desert along the northwesterly transect (Figure 1B).

### Data and processing

#### Vegetation cover changes in the Tibetan plateau

We used the vegetation coverage index (VCI) and leaf area index (LAI) calculated using NOAA CDR of AVHRR (doi: 10.7289/V5PZ56R6 and doi: 10.7289/V5M043BX) to characterise the vegetation cover change of the Tibetan Plateau during the recent four decades (1982–2020). The VCI is an index that can be used to differentiate between green vegetation information and bare soil information at an individual pixel level and is used for estimating the fractional vegetation coverage. Following Zhang et al. (2020), the VCI was calculated from the AVHRR NDVI using the following equation:


$$VCI=\frac{\mathrm{NDVI}-{\mathrm{NDVI}}_{\mathrm{soil}}}{{\mathrm{NDVI}}_{veg}-{\mathrm{NDVI}}_{\mathrm{soil}}}$$

where, NDVI_soil_ is the NDVI value of pure soil pixels, and NDVI_veg_ is the NDVI value of the most vegetated pixel of the image. For the Tibetan Plateau, NDVI_soil_ was the NDVI value with a cumulative frequency of 0.5% in all pixels, and NDVI_veg_ was the NDVI value with a cumulative frequency of 99.5% in all pixels. Only positive VCI values (indicating vegetated land surface) were used in the analysis.

Leaf area index is an index used to measure the number of layers of leaves using remotely sensed data. The LAI data were also derived from the NOAA CDR of AVHRR. For this analysis as well, only positive LAI values (indicating vegetated land surface) were used. Besides, both VCI and LAI in this study had the same spatial resolution as the NOAA CDR of AVHRR data, i.e., with the resolution of 0.05°.

For both VCI and LAI, the changing trends were determined by the methods of least squares regression analysis and *t*-test.

#### Climate dataset

This study collected meteorological data from multiple sources to discern the mechanisms underlying the vegetation responses to warming. Table 1 summarises the meteorological data and their sources. The meteorological data with different spatial and temporal resolutions were first resampled at the same spatial resolution of 0.05° and averaged for the growing (May to October) and non-growing (November to April of the next year) seasons during 2000–2020 (the overlapping time period for all datasets). The vegetation data, VCI and LAI, were also averaged during 2000–2020 to correspond to the climate data. The contour line of 3,000 m in this area was taken as the boundary of the Tibetan Plateau, which was then used to clip all the climate and vegetation maps used in this study.

**Table 1:** Data sets, variables, and their sources.

Variable	Resolution	Source
Air temperature	0.1 arc degrees Monthly	ERA5: ERA5-Land monthly averaged data from 1981 to present (doi: 10.24381/cds.68d2bb30)
Soil surface temperature		
RH		
Soil moisture		
Soil evaporation	500 m 8-day	PML-V2: Penman-Monteith-Leuning Evapotranspiration in Google Earth Engine (Zhang et al., 2017a Kong et al., 2019)
Vegetation transpiration		
Precipitation	0.25 arc degrees	GLDAS-2: NASA Global Land Data Assimilation System Version 2 (doi:10.5067/E7TYRXPJKWOQ)
Snow cover	0.1 arc degrees Monthly	ERA5: ERA5-Land monthly averaged data from 1981 to present (doi: 10.24381/cds.68d2bb30)
Snow depth		
Snow water equivalent		

#### Temperature response curve for vegetation cover

By matching the maps of 2000–2020 averaged VCI and LAI and the growing season air temperature from ERA5 Climate Reanalysis Data (doi: 10.24381/cds.68d2bb30) pixelwise, the multiyear-averaged VCI-temperature and LAI-temperature record was obtained for each pixel. VCI intervals were then generated with a step of 0.005 and the intervals were used to divide the VCI- and LAI-temperature records into different groups. The averaged VCI, LAI, and temperature values and their SDs were calculated in each group and plotted as the final temperature response curves for the vegetation cover indexes. The VCI and LAI threshold values delimiting the decoupling region were identified as the nodes that exhibited the most significant signal loss in the linear regression models in the most and least vegetated areas. The signal losses in the model fitting processes were measured by decreased values of *R*^2^ in the models.

#### Structural equation model

**Figure 2:**
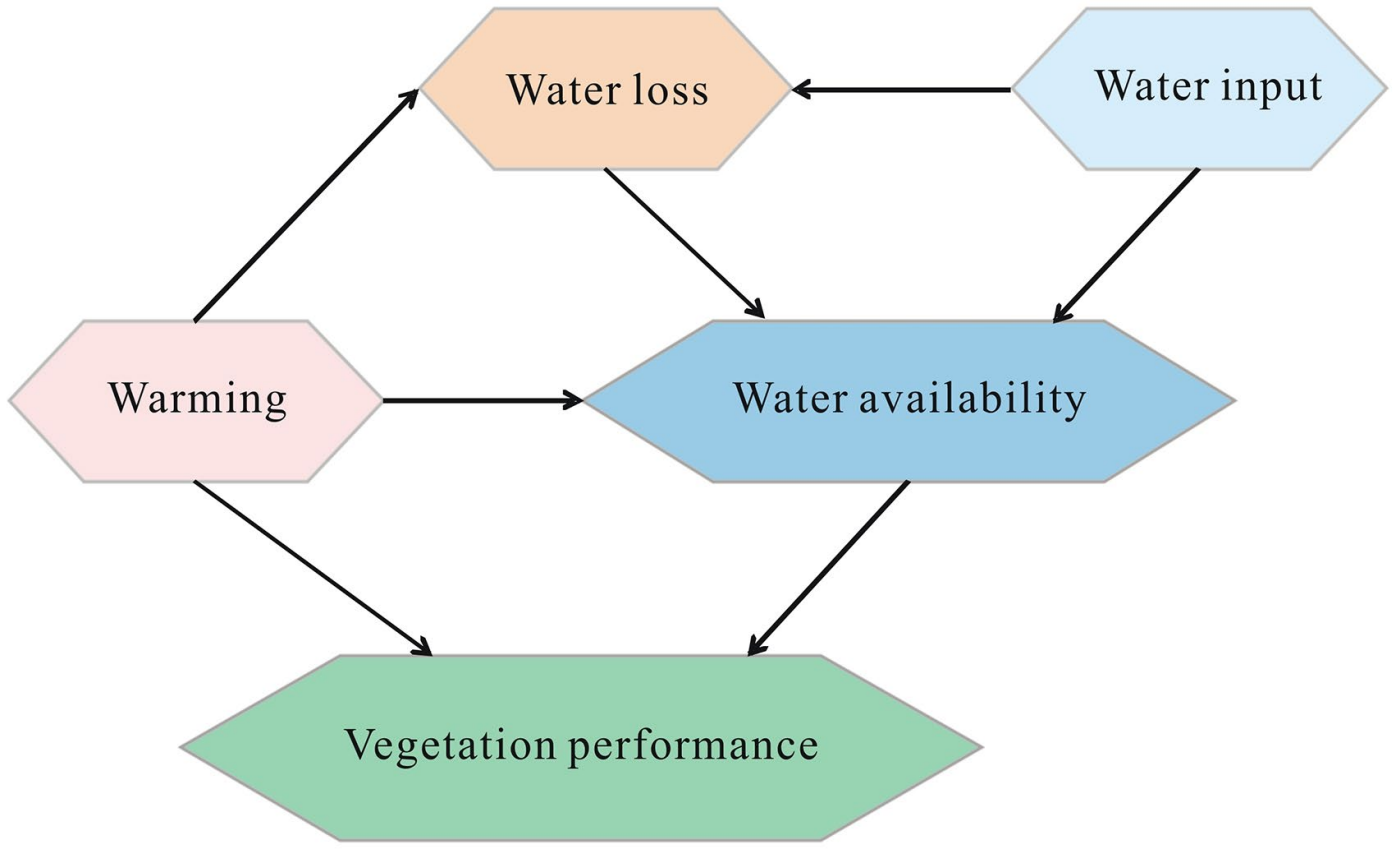
Structural equation meta-model.

In areas where vegetation had been reduced, a structural equation model (Grace and Bollen, 2008) was used to examine the relevance of water and energy variables in plant growth, which was developed based on the ideas embodied in the meta-model, available data, and the principles and procedures laid out by Grace et al. (2010). The meta-model in Figure 2 was established by hypothesising probabilistic expectations based on literature related to the water-energy effects on vegetation (Piao et al., 2014; Sun et al., 2016; Huang et al., 2019). It assumes that vegetation cover is controlled by the temperature and water availability of ecosystems. Water availability is further determined by water loss, reflecting water consumption of ecosystems through evapotranspiration, and the water input, reflecting the water income of ecosystems *via* rain or snow. As evapotranspiration can be influenced by both warming temperatures and water input conditions, “warming” to “water loss” and “water input” to “water loss” were also linked in the meta-model. Furthermore, as the melting of glaciers and permafrost is an important water source for the Tibetan Plateau ecosystems, “warming” was linked to “water availability” in the meta-model.

Indicators for constructs were chosen from the set of available variables and the quantities that could be computed from them (Table 2). These processes were also conducted using the SEM method (Grace and Bollen, 2008; Grace et al., 2010). In these processes, dew point temperature was selected as a measure of water availability in the SEM because dew condensation is an important water resource for plants, especially in semi-arid regions. The model was fitted in the *R* package “blavaan” (Merkle and Rosseel, 2018), estimating the sizes of both the indirect effect size and net effect size of factors such as temperature and water on vegetation cover derived by combining path coefficients.

**Table 2:** Model variables and their indicators^*^.

Model variable	Indicator variables
Vegetation performance	Function of VCI (unitless) and LAI (unitless)
Warming	Function of air temperature (°C) and soil temperature (°C)
Water availability	Function of volumetric soil water (m3/m3) and dewpoint temperature (°C)
Water loss	Function of plant transpiration (m of water equivalent) and soil evaporation (m of water equivalent)
Water input	Function of precipitation (m), snow water equivalent (m of water equivalent), snow cover (%), and snow depth (m)

* Each model variable was combined based on the corresponding environmental variables using an individual structural equation model (Supplementary Figure S3). All model variables were then used to evaluate the integrating structural equation model illustrated in Figure 2.

## Results

### Vegetation cover changes during 1982 to 2020 in the Tibetan plateau

**Figure 3:**
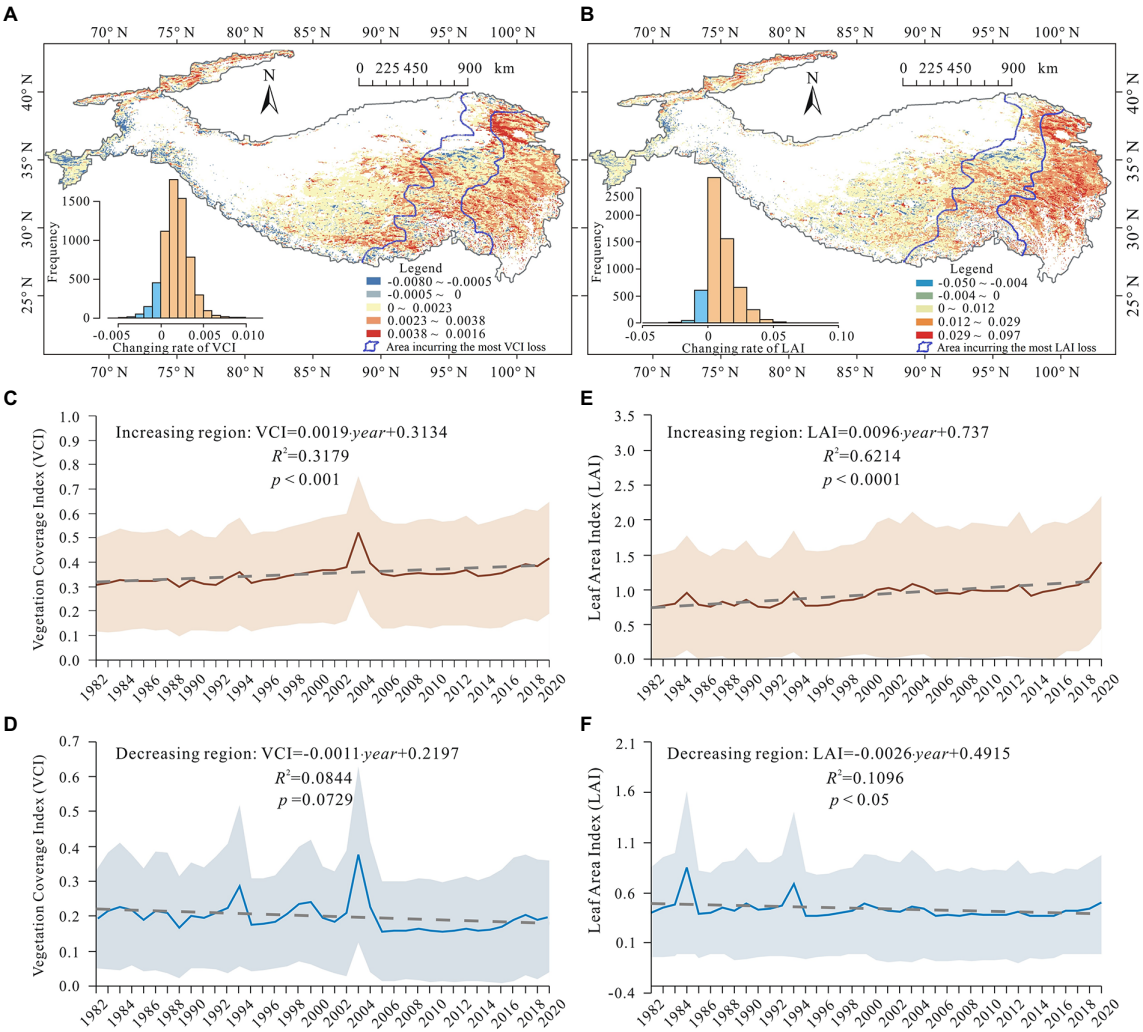
Changes in two vegetation cover indices, vegetation coverage index (VCI), and leaf area index (LAI). **(A)** The maximum VCI trend in the growing seasons during 1982–2020. **(B)** The maximum LAI trend in the growing seasons during 1982–2020. **(C,D)** Changes in the maximum VCI in the growing seasons over the regions showing increasing and decreasing vegetation cover degree during 1982–2020. **(E,F)** Changes in the maximum LAI in the growing season over the regions showing increasing and decreasing vegetation cover during 1982–2020.

As shown in Figure 3, between 1982 and 2020, nearly 89% of the alpine ecosystem in the Tibetan Plateau showed an increasing trend in vegetation cover, with a rate of change of 0.0019 per year for VCI (*p* < 0.001) and 0.0096 per year for LAI (*p* < 0.0001). However, the loss of vegetation cover was observed in the remaining 11% of the alpine ecosystem (marked as dark blue and light blue in Figures 3A,B, respectively), with a significant rate of change (*p* < 0.05) of LAI (*slope* = −0.0026 per year). Spatially, the areas incurring the most vegetation loss are concentrated in the northeast-southwest belt of the central plateau (Figures 3A,B). These areas lie exactly in the middle of the vegetated area of the Tibetan Plateau, where there are transition zones of potential low temperature and drought stresses. This is because as mentioned in the Study area section, elevation inside the plateau increases towards the northwest substantially lowering the temperature, precipitation, and vegetation cover along a northwesterly transect. That is, the detected reduction in the degree of vegetation cover occurred in the moderately vegetated areas but not in the highly vegetated southeast section or the least-vegetated northwest section despite some alleviation of low temperature stress due to the warming climate throughout the plateau. This confirms the existence of divergence in vegetation response to warming in the Tibetan Plateau and reveals its spatial extents.

### Temperature response curves of vegetation cover

**Figure 4:**
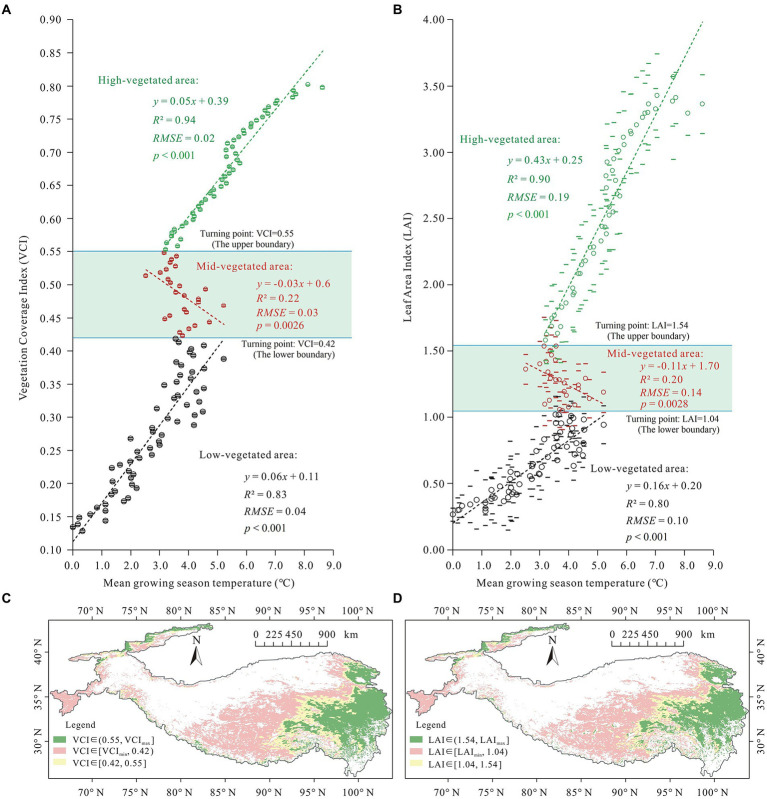
Temperature response curves of vegetation cover on the Tibetan Plateau during the 2000–2020 period. **(A,B)**, Temperature response curves for VCI and LAI, respectively. The averaged VCI and LAI were marked in circles and their SDs were marked *via* bars. **(C,D)** Distribution of vegetation areas on Tibetan Plateau identified using VCI and LAI thresholds shown in **(A,B)**, respectively. The yellow colour depicts regions that had negative correlation of vegetation cover with temperature.

By overlaying maps of vegetation cover indexes (VCI and LAI) that were averaged for 2000–2020 with the corresponding growing season air temperature obtained from ERA5 reanalysis, general temperature response curves of VCI and LAI were generated for the Tibetan Plateau (Figure 4). These findings contradict those of previous studies which revealed the positive responses of vegetation to temperature. Vegetation cover in highly vegetated (VCI < 0.42 and LAI < 1.04) and less vegetated areas (VCI > 0.55 and LAI > 1.54) was positively correlated with the corresponding temperatures during the growing season from May to October. However, in the moderately vegetated area (0.42 < VCI < 0.55 and 1.04 < LAI < 1.54), vegetation cover was found negatively correlated with growing-season temperatures.

Based on the VCI and LAI thresholds that were extracted from the turning points of the vegetation-temperature curves (Figures 4A,B), the moderately vegetated areas where vegetation cover was negatively correlated with temperature were identified, which were marked in yellow in Figures 4C,D. The areas primarily overlapped with areas exhibiting a reduction in vegetation cover that as depicted in Figure 3, implying that the latter reflects the negative response of vegetation to warming. In addition, in a field survey undertaken on the south-eastern Tibetan Plateau from May 13 to May 20, 2021 during the late spring/early summer growing period, extensive birch (Betula platyphylla Suk.) die-back was observed in the moderately vegetated areas (Supplementary Figure S1). There were no indications of pest or fungal infestations were found on either dead or living trees. This observation provides evidence for the degrading trend detected using remote sensing, as well as the decoupling that was revealed by temperature response curves.

### Mechanisms underlying the decoupling responses of vegetation to warming

**Figure 5:**
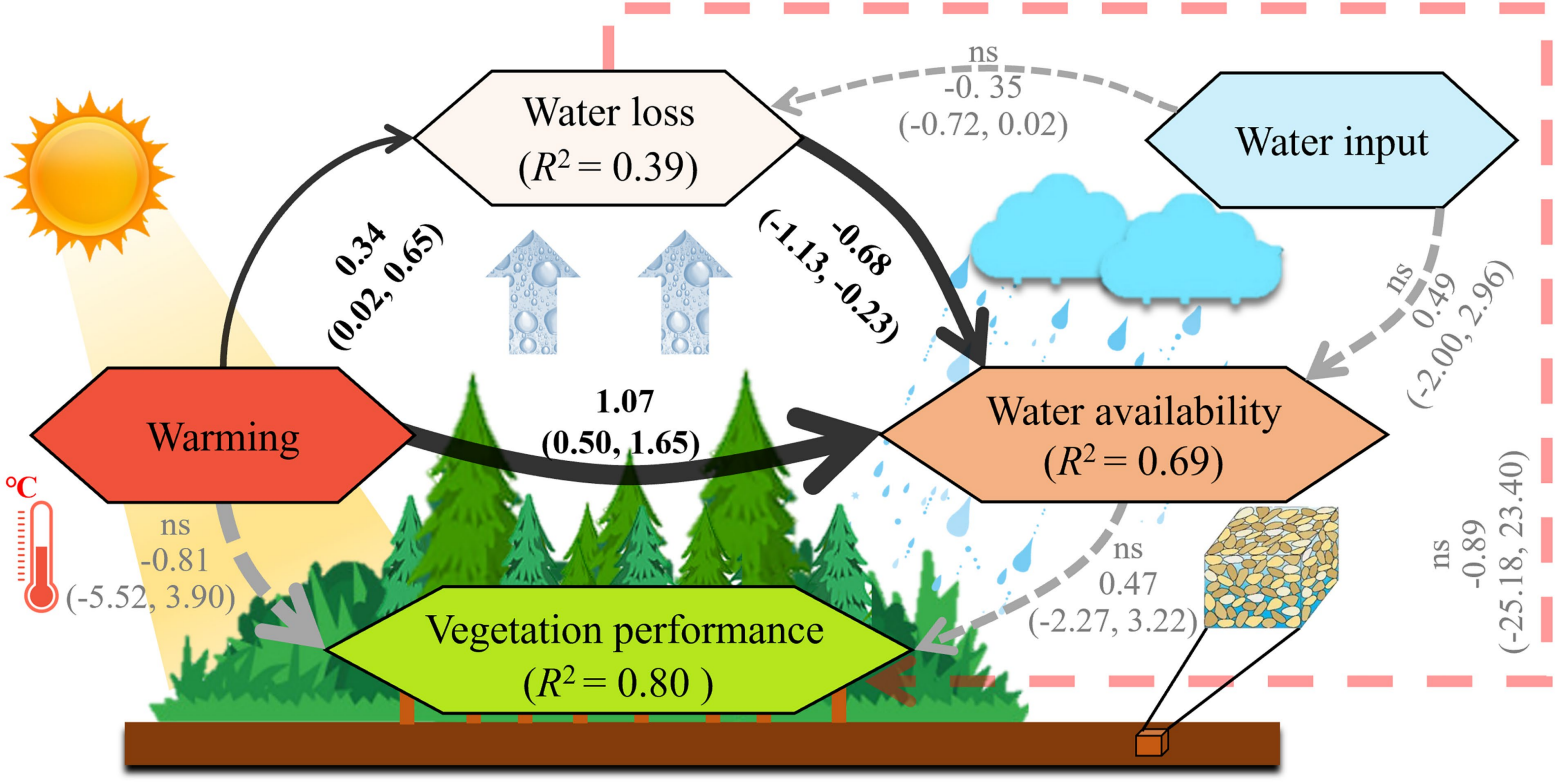
Structural equation model supported by the data for the degraded vegetation areas in the Tibetan Plateau. Solid black arrows represent significant effects (the 95% credible interval does not include 0) and gray dashed arrows represent non-significant effects (the 95% credible interval includes 0). The pink line represents the newly added linkage from the fitting processes, which was not included in the initial meta-model. Arrow thickness is proportional to path factor size. The test statistic was: *MargLogLik* = −102.02 and *BIC* = 59.01, with 25 model degrees of freedom and *PPP* = 0.45, indicating a close fit for the model data.

In areas where vegetation had been reduced, the established structural equation meta-model in Figure 2 was evaluated using multiple remote sensing and reanalysis data listed in Tables 1, 2. The evaluated structural equation model (SEM) supported by the data was shown in Figure 5.

As shown in Figure 5, there is clear evidence that warming increases water loss from the ecosystem (standardised path coefficient 0.34; 95% CI [0.02, 0.65]), as measured using variables related to evapotranspiration. In addition, water loss decreased water availability of the ecosystem (coefficient −0.68; 95% CI [−1.13, −0.23]), which was measured mainly by variables related with soil moisture content. Warming therefore has an indirect negative effect on ecosystem water availability that was mediated by water loss (coefficient −0.23). This feedback indicates that the negative vegetation–temperature relationship occurred because of decreasing water availability as the consequence of increased evapotranspiration. Most notably, these two linkages that support this feedback, namely, the “warming” to “water loss” link and the “water loss” to “water availability” link, are two of the three most significant linkages of the SEM. This highlights their key role in driving the decoupling phenomenon in areas that are becoming degraded.

“Warming” to “water availability” is the other significant link in the SEM in Figure 5 (coefficient 1.07; 95% CI [0.50, 1.65]). This implied the presence of a strong positive effect of warming on water availability in the Tibetan Plateau, which is even greater than the positive effect of water input variables on water availability (coefficient 0.49, ns). These findings suggest that melting of glaciers and permafrost is more important than precipitation in determining water availability for ecosystems in this area. This also explains why the negative vegetation response to warming is limited to a relatively small area of the Tibetan Plateau. This is because the drying effect of warming on vegetation by increased evapotranspiration can be partially offset in warmer climates due to an increase in the available meltwater. Further, Figure 5 shows that the independent effects of both warming (coefficient −0.81, ns) and water availability (coefficient 0.47, ns) on vegetation cover in the moderately vegetated region were non-significant. This is because both temperature and water availability are strong limiting factors for vegetation growth in this area. Their interactions dominate the vegetation growth, but individually they do not have decisive effects. These results also demonstrate that failure to account for variation in water availability driven by evapotranspiration fluctuations would make it difficult to explain the negative influences of warming on vegetation.

In addition, as shown in Figure 5, there is a weak but direct inhibitory effect of water loss on vegetation cover (coefficient −0.89, ns) that was supported by the data. This linkage (marked with the pink dashed line) was not included in the initial meta-model but was subsequently added during the fitting processes. This suggests that ecosystem drying may limit vegetation growth in other ways, which were not included in the meta model. It is likely that dry environmental conditions may affect vegetation growth by reducing microorganism activity, soil nutrient availability, and other factors. However, these influences and their interactions were not included in this study due to the difficulty in assessing them using remote sensing and reanalysed data. In addition, the final model did not make any simplifications in the meta-model because it would otherwise fail to detect previously detected pathways and result in a notable loss of signal, as indicated by the reduced values of *R*^2^ in the model.

## Discussion

### How hydrological variables mediate responses to warming by vegetation

**Figure 6:**
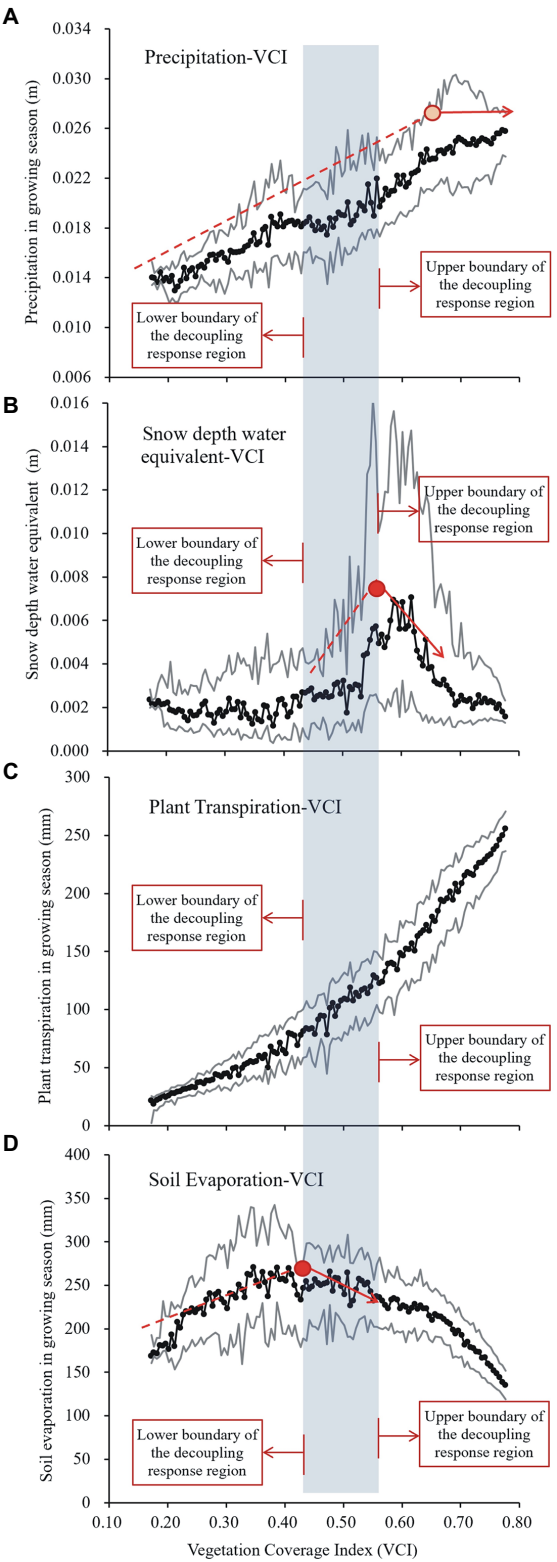
Corresponding relationship between the tipping points for the vegetation coverage index (VCI) response to multiple water variables, along with the upper and lower boundaries for areas where vegetation growth responds negatively to warming. **(A)** Precipitation vs. VCI. **(B)** Snow water equivalent vs. VCI. **(C)** Plant transpiration vs. VCI. **(D)** Soil evaporation vs. VCI. Black lines with black dots indicate the mean values for the corresponding water variables at each VCI interval. Grey lines indicate the mean plus/minus SDs of the corresponding water variables in each VCI interval. Red dots indicate the tipping points for the VCI response to the corresponding water variables.

The relationships between the level of vegetation cover and variables affecting water availability on the Tibetan Plateau was shown in Figure 6, which reveals how the level of vegetation cover varied in response to changes in key hydrological variables and therefore their role in mediating warming responses by the vegetation. In Figure 6, VCI was selected as being a representative index to explore the response of vegetation cover to water variables on the plateau. This can reduce redundancy because the VCI and LAI values for the plateau show high levels of similarity.

The decoupling region that is delimited by the two VCI thresholds, namely, the upper boundary of VCI = 0.55 and the lower boundary of VCI = 0.42, is shaded in light blue in Figure 6. The upper boundary is adjacent to the saturation point for vegetation response to growing season precipitation (Figure 6A). Besides, it is almost consistent with the turning point for vegetation response to the snow water equivalent (Figure 6B). These phenomena can be interpreted as follows: warming induces vegetation loss through temperature-induced ET increase; yet when water availability is not limited, the increased ET could not inhibit vegetation growth. This explains why the decoupling occurred in the central plateau but not in the wet southeastern plateau. Moreover, Figures 6C,D show that the lower boundary of the decoupling response for vegetation to temperature is observed when soil evaporation begins to decrease as vegetation cover increases. As soil evaporation decreases with increases in vegetation cover in areas with a certain degree of vegetation cover, such as a predominantly closed canopy or a high-density grassland, findings reveal that the effect of warming in reducing water availability and inhibiting vegetation growth by increasing evapotranspiration only occurs in areas with a certain vegetation density (VCI > 0.42 or LAI > 1.04 for the Tibetan Plateau). This explains why the decoupling did not occur in dry areas with lower levels of vegetation cover in the plateau (i.e., the northwest areas of the Tibetan Plateau), but occurred in moderately vegetated areas in the central plateau. Overall, these findings revealed the hydrological conditions that result in the observed negative response for vegetation to warming. That can be identified as determining conditions for negative responses in vegetation to temperature in the Tibetan Plateau.

**Figure 7:**
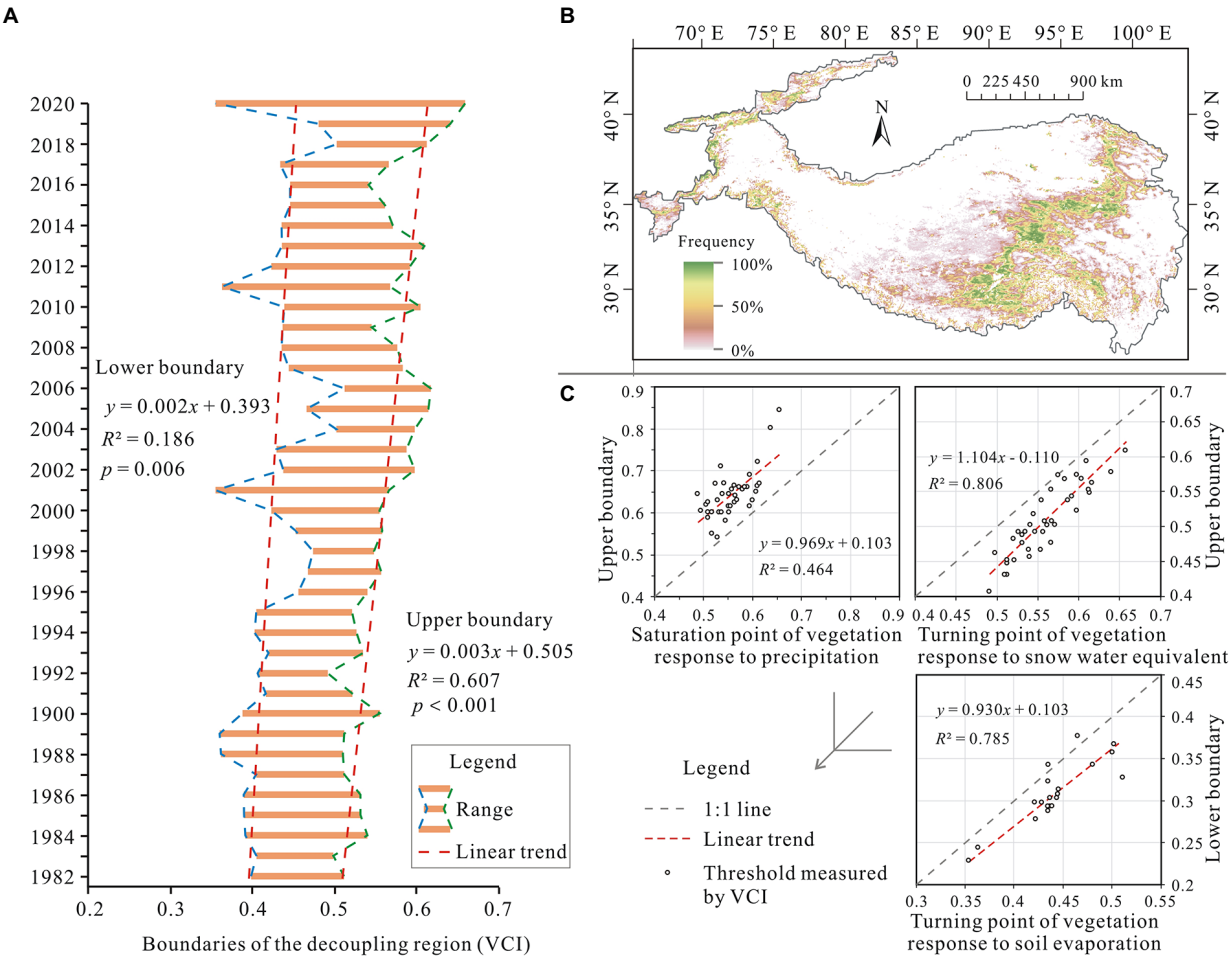
Spatiotemporal variations for the decoupling region and their relationships with changes in the tipping points of the VCI response to multiple water variables. **(A)** Boundary variations for the decoupling region over the last 40 years. **(B)** Spatial extent changes of the decoupling region expressed by frequency. **(C)** Relationships between the changes in the decoupling boundary and the turning point changes in the VCI response to precipitation, snow water equivalent, and soil evaporation.

To test whether the observed determining conditions for the decoupling phenomenon are real, spatiotemporal variations were analyzed for the decoupling region from 1982 to 2020, as well as their consistency with changes to these tipping points for the VCI response to these key hydrological variables (Figure 7). As shown in Figure 7A, the VCI thresholds for both the upper and lower boundaries of the decoupling region increased from 1982 to 2020 with rates of change of 0.002 and 0.003 per year for the lower and upper boundaries, respectively. This suggests that the decoupling region has shifted to the southeast over the last 40 years. However, the shifting extent of the decoupling region has been minimal with a frequency of occurrence of >80%, which has been concentrated in the narrow northeast-southwest belt of the central plateau (Figure 7B). In addition, Figure 7C shows that the spatiotemporal variations in the decoupling region can be explained by changes in tipping points for the VCI response to key hydrological variables. The explanatory degrees of two water input terms, namely precipitation and snow water equivalent, to the upper boundary variations for the decoupling region were approximately 47 and 80%, respectively. The water consumption term, i.e., the turning point of decreasing soil evaporation with increasing vegetation cover (representing the proportion of plant transpiration in total evapotranspiration), explained approximately 79% of the lower boundary fluctuation.

### Reasons for spatiotemporal variations of the decoupling region from 1982 to 2020

Hydrological changes reported by previous studies (Supplementary Figure S2) have confirmed the observed hydrology-driven variations in the decoupling region (Berry and Bjorkman, 1980; Grace et al., 2010; Xiao et al., 2016; Deng and Zhang, 2018). Specifically, precipitation in the Tibetan Plateau is influenced by westerly winds (mainly controlling the northwest part of the plateau) and Asian monsoons (mainly controlling the southeast part of the plateau; Grace et al., 2010). In recent decades, westerly winds over the plateau have escalated, and transport a greater amount of water vapour, whereas Asian monsoons have weakened, thus transporting less water vapour to the region than they did previously (Berry and Bjorkman, 1980; Deng and Zhang, 2018). Therefore, precipitation in the Tibetan Plateau increased in the northwest but decreased in the southeast in recent decades (Supplementary Figure S2A; Xiao et al., 2016). Simultaneously, evapotranspiration has increased throughout the plateau due to the overall increase in the temperature (Supplementary Figure S2B; D’Arrigo et al., 2004; Yang et al., 2011; Beier et al., 2012). However, as vegetation cover was sparser in the northwest than that in the southeast, the increase in evapotranspiration in the northwest was smaller than that in the southeast (Fan and Cheng, 2002). Consequently, on the northern and western regions of the plateau, the substantial increase in precipitation and the slight increase in evapotranspiration have resulted in a net increase of plant-available water, thereby alleviating water stress (Supplementary Figure S2C; Xiao et al., 2016). This has caused the south-eastward movement of the lower boundary of the decoupling region. On the southern and eastern regions of the plateau, precipitation has, however, decreased while evapotranspiration increased. This has depleted water resources and exacerbated water stress (Supplementary Figure S2C; Xiao et al., 2016). Consequently, the upper boundary has also moved towards the southeast. In addition, increasing meltwater from glaciers and permafrost due to rising temperatures (Supplementary Figure S2D) has also promoted the south-eastward shift of the lower boundary. The north-western plateau is an endorheic basin and the increasing meltwater remained in the ecosystem to a large extent and has improved water availability for the vegetation (Grace and Bollen, 2008; Zhang et al., 2019a, 2020). However, the increased meltwater is unlikely to have relieved vegetation-water stress in the south-eastern region of the plateau. This is because it is an exogenous region and the influence of increasing meltwater has decreased due to increasing runoff (Supplementary Figure S2E; Zhang et al., 2017a; Yao et al., 2019). Findings from the current study, together with the reported variations for key hydrological variables on the Tibetan Plateau, suggest that the decoupling phenomenon of vegetation responses to warming with respect to water availability is plausible. Changes in the extent of the decoupling region on the Tibetan Plateau depend on changes in its hydrological conditions.

## Conclusion

This study found reductions in vegetation coverage and decoupling of vegetation growth to warming in the moderately vegetated areas of the Tibetan Plateau. An integrated SEM elucidated the primary mechanisms underlying the decoupling phenomenon and demonstrated that the effect of temperature rise on vegetation growth becomes complex due to the influence of water availability. These findings prompt us to re-examine the longstanding paradigm that vegetation growth in alpine ecosystems is limited by temperature and that warming is therefore conducive to increased vegetation coverage. If our results are extrapolated to examine effects of future warming over the coming decades, it is therefore hypothesised that further warming could lead to substantial vegetation loss in semi-arid ecosystems with certain levels of vegetation cover. This reduces the capacity of the vegetation to sequester CO_2_, thereby ultimately accelerating climate change. In addition, the decoupling of the positive relationship between vegetation and temperature in water-limited alpine ecosystems has suggested that the previously reported warming-induced increase in vegetation growth would weaken (Smith et al., 2016; Mercado et al., 2018). This is consistent with recent studies that have demonstrated that recent global vegetation changes were far more due to CO_2_ fertilisation than because of climate change (Piao et al., 2020).

Furthermore, findings of the current study will add further nuances to the prediction of changes in vegetation in alpine ecosystems in a warming world. Increasing evapotranspiration that intensifies plant drought stress reduces vegetation cover with rising temperatures in water-limited alpine ecosystems. Warming in these ecosystems, however, will lead to accelerated thawing of glaciers and permafrost (Wang et al., 2019b). This increases liquid water in these ecosystems and alleviates the negative impact of increased temperature on vegetation by increasing evapotranspiration. Interactions between these two feedbacks have resulted in a shift, but not an extension of the decoupling zone on the Tibetan Plateau over the last 4 decades. However, with meltwater from glaciers and permafrost running dry in the future, the influence of increasing evapotranspiration would prevail, resulting in an expansion of the decoupling zone. Findings from the study have highlighted the increase in the risk of degrading vegetation covering large spatial area in the future with accelerating or decelerating warming rates. The current vegetation degradation maps developed during this study can also aid planning and prioritisation of the most vulnerable areas for protection and climate change mitigation.

## Data availability statement

The original contributions presented in the study are included in the article/Supplementary material, further inquiries can be directed to the corresponding authors.

## Author contributions

ZY and JX conceived the study. XD and WZ prepared the data, set up the model, and conducted statistical analysis and drew inferences. ZY and AM further improved the analysis design. All authors were involved in the writing and editing of the manuscript. All authors contributed to the article and approved the submitted version.

## Funding

We would like to thank the National Key R&D Program of China (Grant No. 2016YFC0402710), National Natural Science Foundation of China (Grant No. U2240217, 51539003 and 41761134090), National Science Funds for Creative Research Groups of China (No. 51421006), and the program of Dual Innovative Research Team in Jiangsu Province and the Special Fund of State Key Laboratory of Hydrology-Water Resources and Hydraulic Engineering (Grant No. 20145027312). AM was supported by the US Department of Energy TES grant DE-SC0020116 and the US National Science Foundation EAR CAREER award (Grant No. 2046768).

## Conflict of interest

The authors declare that the research was conducted in the absence of any commercial or financial relationships that could be construed as a potential conflict of interest.

## Publisher’s note

All claims expressed in this article are solely those of the authors and do not necessarily represent those of their affiliated organizations, or those of the publisher, the editors and the reviewers. Any product that may be evaluated in this article, or claim that may be made by its manufacturer, is not guaranteed or endorsed by the publisher.

## Supplementary material

SUPPLEMENTARY FIGURE S1 Observed extensive forest with sporadic dead birch (*Betula platyphylla* Suk.) in Jiangda County during the field survey. **(A)** Reconstructed image of the hillside from 292 photos taken using an unmanned aerial vehicle (UAV). **(B,C)** Vertical view from the UAV at camera locations P1 and P2. **(D)** Horizontal view from the foot of the mountain at camera location P3.

SUPPLEMENTARY FIGURE S2 Changes in multiple hydrological variables in the Tibetan Plateau reported by previous studies. **(A)** Precipitation changes (from Zhao et al., 2019). **(B)** Evapotranspiration changes (based on the data of Zhang et al., 2019a). **(C)** Soil moisture changes (from Zhao et al., 2019). **(D)** Glacier changes (from Wang et al., 2019a). **(E)** Runoff changes (from Zhang et al., 2019b).

SUPPLEMENTARY FIGURE S3 Construction of the model variables (i.e., latent variables of the SEM). **(A)** Water loss. **(B)** Water input. **(C)** Warming. **(D)** Water availability. GS and NGS stand for growing season and non-growing season, respectively.
