# Supplementary figures and images for: Increasing evapotranspiration decouples the positive correlation between vegetation cover and warming in the Tibetan plateau
Source: Front Plant Sci. 2022 Sep 23;13:974745. doi: 10.3389/fpls.2022.974745 (PMC9537816; doi:10.3389/fpls.2022.974745)

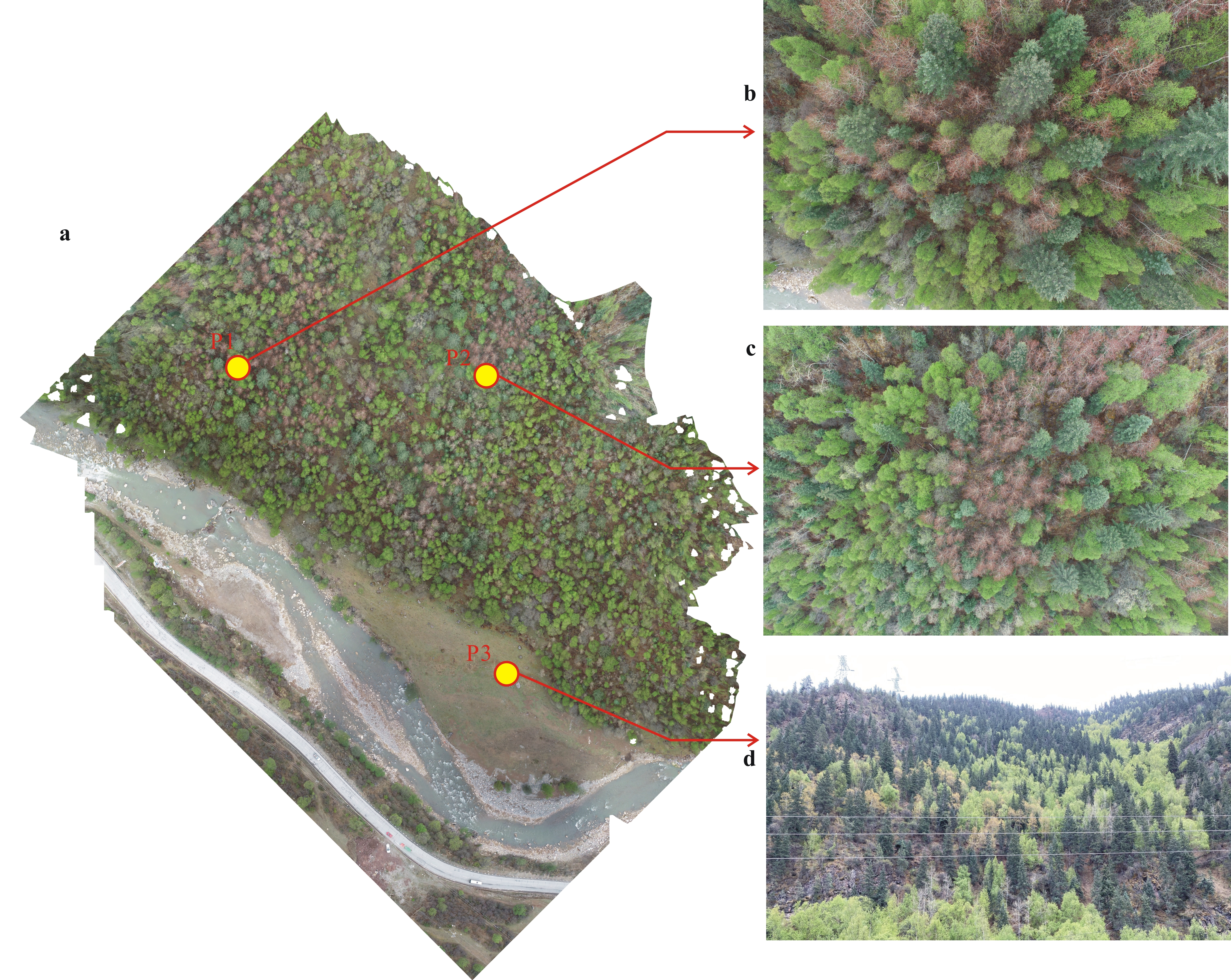

Supplement: SUPPLEMENTARY FIGURE S1 — Observed extensive forest with sporadic dead birch (Betula platyphylla Suk.) in Jiangda County during the field survey. (A) Reconstructed image of the hillside from 292 photos taken using an unmanned aerial vehicle (UAV). (B,C) Vertical view from the UAV at camera locations P1 and P2. (D) Horizontal view from the foot of the mountain at camera location P3. [file Image_1.TIF]

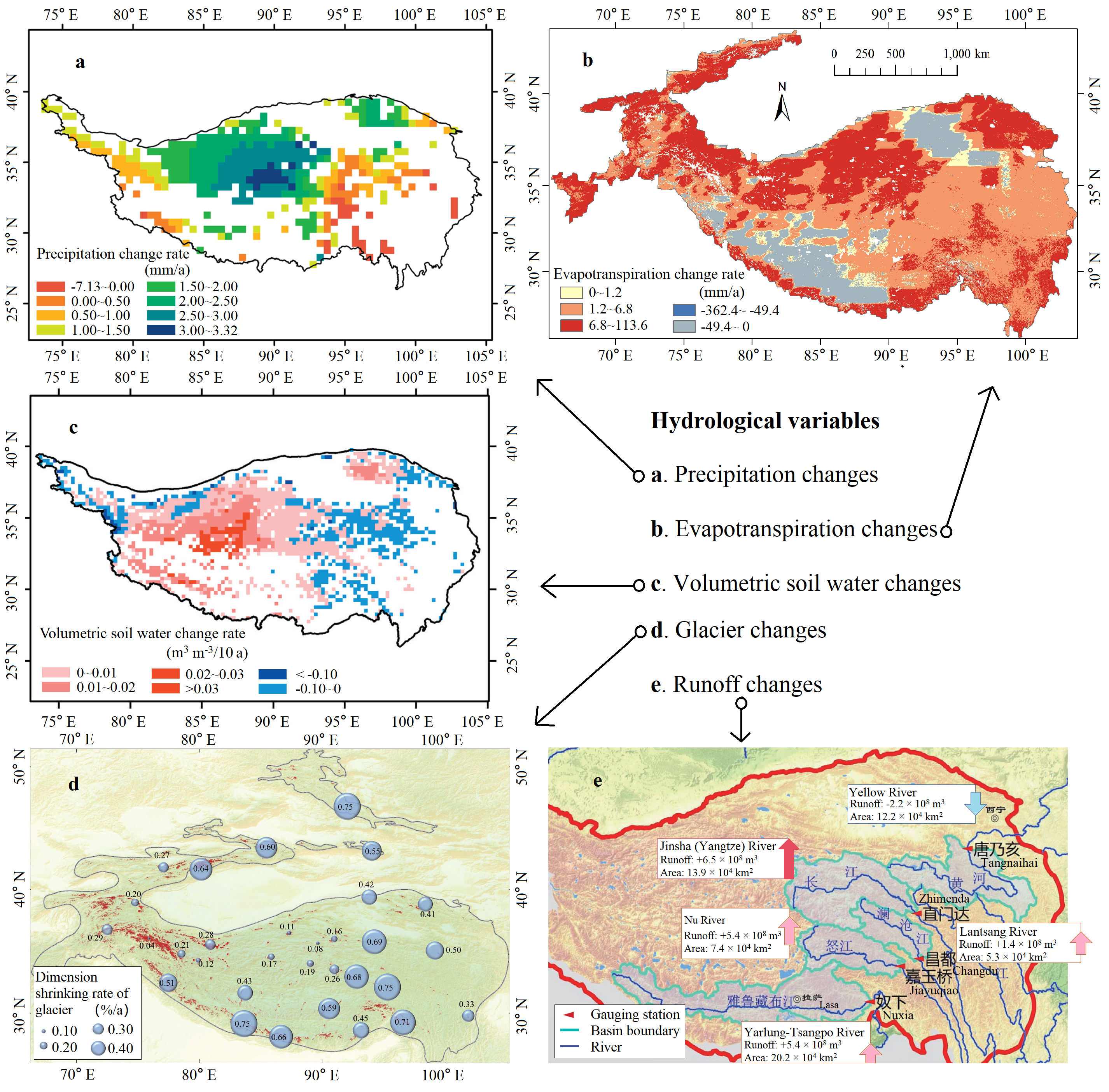

Supplement: SUPPLEMENTARY FIGURE S2 — Changes in multiple hydrological variables in the Tibetan Plateau reported by previous studies. (A) Precipitation changes (from Zhao et al., 2019). (B) Evapotranspiration changes (based on the data of Zhang et al., 2019a). (C) Soil moisture changes (from Zhao et al., 2019). (D) Glacier changes (from Wang et al., 2019a). (E) Runoff changes (from Zhang et al., 2019b). [file Image_2.TIF]

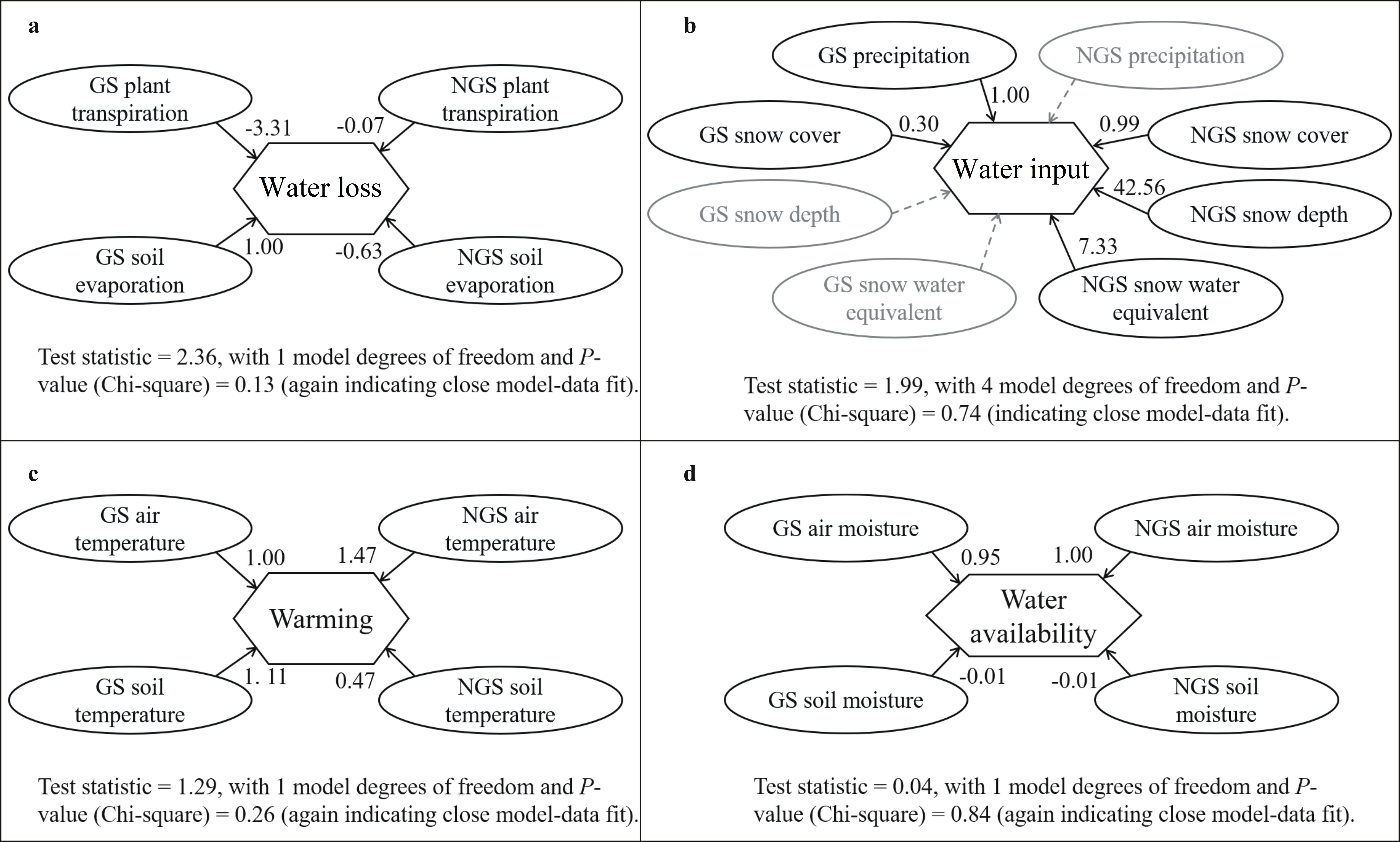

Supplement: SUPPLEMENTARY FIGURE S3 — Construction of the model variables (i.e., latent variables of the SEM). (A) Water loss. (B) Water input. (C) Warming. (D) Water availability. GS and NGS stand for growing season and non-growing season, respectively. [file Image_3.TIF]
